# Solvatochromic Study of 2‑(*N*,*N*‑dimethyl)-3-alkynyl-pyridine Fluorophores

**DOI:** 10.1021/acsomega.6c03632

**Published:** 2026-06-22

**Authors:** Leah Gross, Aimee Phillips, Lavender Allen, Zane Burnett, Korinna Welch, Prerna Masih, Chandra Prayaga, Tanay Kesharwani, Aaron Wade

**Affiliations:** † Department of Physics, 6491 University of West Florida, Pensacola, Florida 32514-5750, United States; ‡ Department of Biology, 1263 Bard College, Annandale-on-Hudson, New York 12504-5000, United States; § Department of Chemistry, 1263 Bard College, Annandale-on-Hudson, New York 12504-5000, United States

## Abstract

A series of pyridine-based fluorophores—2-(*N*,*N*-dimethyl)-3-alkynyl-pyridine (NNDAP), 4-[2-[2-(dimethylamino)-3-pyridyl]­ethynyl]­anisole (NNDAP-OMe), and 4-[2-[2-(dimethylamino)-3-pyridyl]­ethynyl]­benzonitrile (NNDAP-CN)—were synthesized and their solvent-dependent photophysical properties were systematically investigated. Solvatochromic shifts in absorption and emission spectra were analyzed using the Bilot–Kawski, Lippert–Mataga, Bakhshiev, and Reichardt correlation methods to estimate ground- and first excited-state electric dipole moments. All compounds exhibit increased polarity in the excited state, with NNDAP-CN showing the largest change in dipole moment upon excitation. Solute–solvent interactions were also examined using the Kamlet–Taft and Catalán multiparameter linear solvation energy relationship models, revealing that solvent dipolarity/polarizability interactions influence absorption shifts, while emission shifts are influenced by a combination of solvent dipolarity/polarizability and solvent basicity. All fluorophores readily permeate HeLa cells and exhibit stable blue fluorescence localized primarily in the perinuclear cytoplasmic region, supporting their potential application as environment-sensitive fluorescent probes.

## Graphical abstract

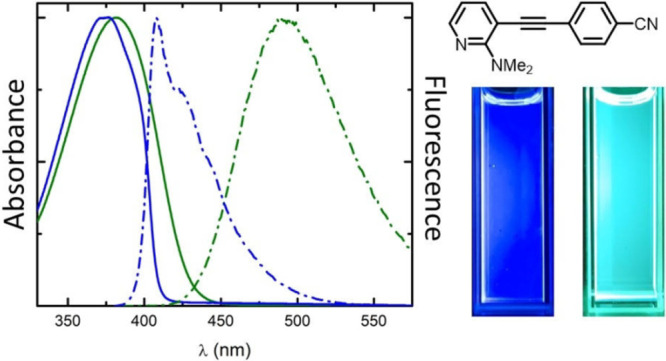

## Introduction

Small-molecule organic fluorophores are widely used across biology, chemistry, and materials science due to their tunable photophysical properties and broad applicability in chemical sensing, fluorescence microscopy, organic electronics, photovoltaics, and nonlinear optical materials.^1–6^ Among these, nitrogen-containing heterocycles such as pyridine and quinoline represent an important class of chromophores because of their structural versatility, electronic tunability, and established roles in biologically active compounds.^7–12^ Pyridine-based scaffolds, in particular, have been extensively explored as fluorescent probes and sensors, owing to their sensitivity to local chemical environments and their ability to engage in specific intermolecular interactions.^13–17^

Pyridine-based ligands have also been employed in electrochemical biosensors, where metal–ligand coordination plays a critical role in signal transduction.^18^ Despite these advances, the mechanisms are not fully understood, but understanding how they work is essential for rational design. As a result, a detailed understanding of how solvent polarity and specific solute–solvent interactions influence the electronic structure and excited-state behavior of such fluorophores remains essential for rational probe design, particularly for applications involving heterogeneous or biological environments.

Solvatochromism—the dependence of electronic absorption and emission spectra on solvent properties—provides a powerful experimental approach for probing changes in molecular dipole moments and solute–solvent interactions upon electronic excitation.^19–24^ Classical single-parameter models, including the Bilot–Kawski,^25–28^ Lippert–Mataga,^29–31^ and Bakhshiev,^32,33^ correlations, are rooted in Onsager’s reaction field theory and relate spectral shifts to the solvent dielectric constant (ϵ) and the solvent refractive index (*n*).^30,32,34^ Because each solvatochromic model incorporates slightly different treatments of solvent reaction fields and spectral shifts, comparison of multiple formalisms allows for a better estimation of the excited-state dipole moments. The Lippert–Mataga approach offers a convenient estimate of the change in dipole moment Δμ upon excitation but can overestimate it as it neglects solute polarizability. The Bakhshiev method partially corrects for this limitation and generally yields values, for the change in the dipole moment, in closer agreement with Bilot–Kawski analysis, while the latter uniquely enables independent determination of ground- and excited-state dipole moments. These models primarily capture nonspecific electrostatic interactions between the solute and solvent, while the Reichardt approach introduces an empirical solvent polarity parameter­(*E*_*T*_^*N*^) to account for short-range interactions, making it particularly useful when specific interactions play a significant role.^24,35^

To further disentangle the contributions of specific solute–solvent interactions, multiparameter linear solvation energy relationship (LSER) models such as those developed by Kamlet–Taft^36^ and Catalán^37^ have been widely employed. These approaches explicitly consider solvent dipolarity, polarizability, and hydrogen-bonding interactions, enabling a more detailed interpretation of the mechanisms governing spectral shifts. In particular, the Catalán model separates solvent dipolarity and polarizability into independent parameters, providing additional insight into excited-state charge redistribution and specific solvent effects.

In this work, a series of pyridine-based fluorophores—2-(*N*,*N*-dimethyl)-3-alkynyl-pyridine (NNDAP), 4-[2-[2-(dimethylamino)-3-pyridyl]­ethynyl]­anisole (NNDAP-OMe), and 4-[2-[2-(dimethylamino)-3-pyridyl]­ethynyl]­benzonitrile (NNDAP-CN)—were synthesized and systematically investigated. The effects of solvent environment on their absorption and emission spectra were analyzed using both classical solvatochromic models and multiparameter LSER methods to estimate ground- and excited-state dipole moments and to elucidate the nature of solute–solvent interactions. In addition, the changes to the base molecule by the addition of the substituents (−CN or −OMe) provide a convenient strategy for tuning excited-state charge redistribution and solvent sensitivity in donor–acceptor fluorophores. In addition, the cellular uptake and fluorescence behavior of these compounds were evaluated in HeLa cells to assess their suitability for fluorescence imaging applications.

## Experimental Section

### Synthesis

The fluorophores 2-(*N*,*N*-dimethyl)-3-alkynyl-pyridine (NNDAP), 4-[2-[2-(dimethylamino)-3-pyridyl]­ethynyl]­anisole (NNDAP-OMe), and 4-[2-[2-(dimethylamino)-3-pyridyl]­ethynyl]­benzonitrile (NNDAP-CN) were synthesized via a two-step procedure. In the first step, nucleophilic aromatic substitution of 2-fluoro-3-iodopyridine with dimethylamine was carried out using dimethylformamide (DMF) as the solvent. The resulting intermediate was subsequently subjected to palladium-catalyzed cross-coupling reactions with phenylacetylene, 4-methoxyphenylacetylene, or 4-cyanophenylacetylene to yield the corresponding alkynyl-substituted pyridine derivatives (Figure 1). Detailed synthetic procedures and characterization data are provided in the Supporting Information.

**Figure 1:**
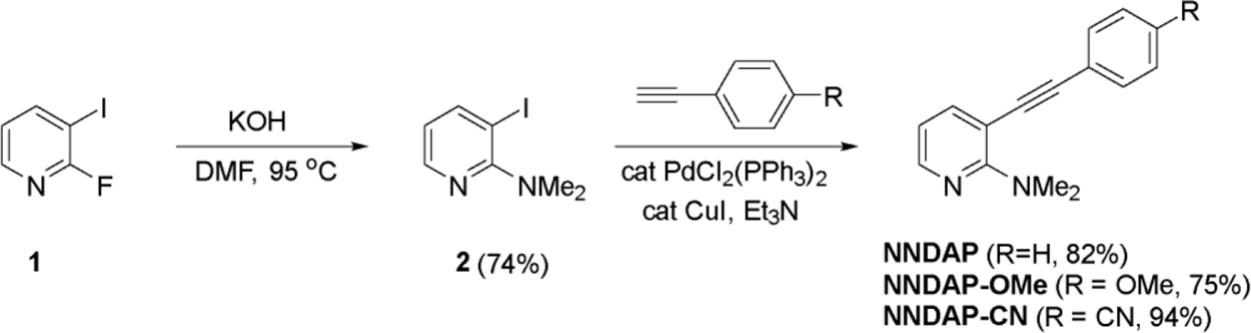
Synthesis of novel pyridine-based fluorophores.

### Spectral Measurements

Solutions of NNDAP, NNDAP-OMe, and NNDAP-CN were prepared in ten different solvents at a concentration of 5.78 × 10^–5^ M. Samples were placed in sealed 1.0 cm quartz cuvettes to minimize solvent evaporation during measurements. UV–vis absorption spectra were recorded using a Shimadzu UV-2600 spectrophotometer and fluorescence emission spectra were obtained using a Cary Eclipse fluorescence spectrophotometer, with excitation at the wavelength corresponding to the lowest-energy absorption maximum for each solvent. Fluorescence quantum yields were measured using an RF-600 spectrofluorometer equipped with an integrating sphere. Sample densities were determined using a Quantachrome UltraPyc 1200e gas pycnometer for solid samples and a microsyringe and analytical balance for liquid samples. The Onsager radius was calculated for each compound and found to be 4.29 Å.

### Fluorescence Visualization in HeLa Cells

HeLa cells were seeded at a density of 2 × 10^4^ cells per well in 24-well plates and cultured for 48 h prior to imaging. Fluorophore stock solutions were prepared in dimethyl sulfoxide (40.94 mg·mL^–1^) and diluted with phosphate-buffered saline to a final concentration of 100 μg·mL^–1^. Following PBS washing, cells were incubated with each fluorophore for 15 min at room temperature. Fluorescence imaging was performed using an EVOS M5000 fluorescence microscope (Thermo Fisher Scientific) equipped with excitation and emission filters centered at approximately 357 and 447 nm, respectively. Phase-contrast, fluorescence, and merged images were collected at 10× magnification. All three compounds readily permeated the cellular membrane and produced detectable intracellular fluorescence after incubation.

## Results and Discussion

### Density Functional Theory Calculations

Density functional theory calculations were performed using Spartan 18 demonstrate that electron-donating and electron-withdrawing molecules systematically modulate the frontier orbital energies within the NNDAP series, Figure 2. NNDAP-OMe exhibits a HOMO energy of −5.5 eV and a LUMO energy of −1.5 eV, corresponding to a calculated HOMO–LUMO gap of 4.0 eV. The unsubstituted NNDAP displays slightly stabilized orbitals, with a HOMO at −5.6 eV and a LUMO at −1.7 eV, yielding a gap of 3.9 eV. In contrast, NNDAP-CN shows substantial stabilization of both frontier orbitals, with a HOMO of −6.0 eV and a LUMO of −2.4 eV, resulting in a reduced gap of 3.6 eV. The pronounced lowering of the LUMO energy in NNDAP-CN reflects the strong electron-withdrawing character of the nitrile substituent, which enhances π-acceptor strength and promotes intramolecular charge-transfer character. Although both orbitals are stabilized in the cyano derivative, the greater relative stabilization of the LUMO leads to a narrowed energy gap, consistent with the experimentally observed red-shifted absorption and enhanced solvatochromic response. These trends indicate that substituent-induced electronic effects systematically tune excited-state energetics and charge separation across the fluorophore series.

**Figure 2:**
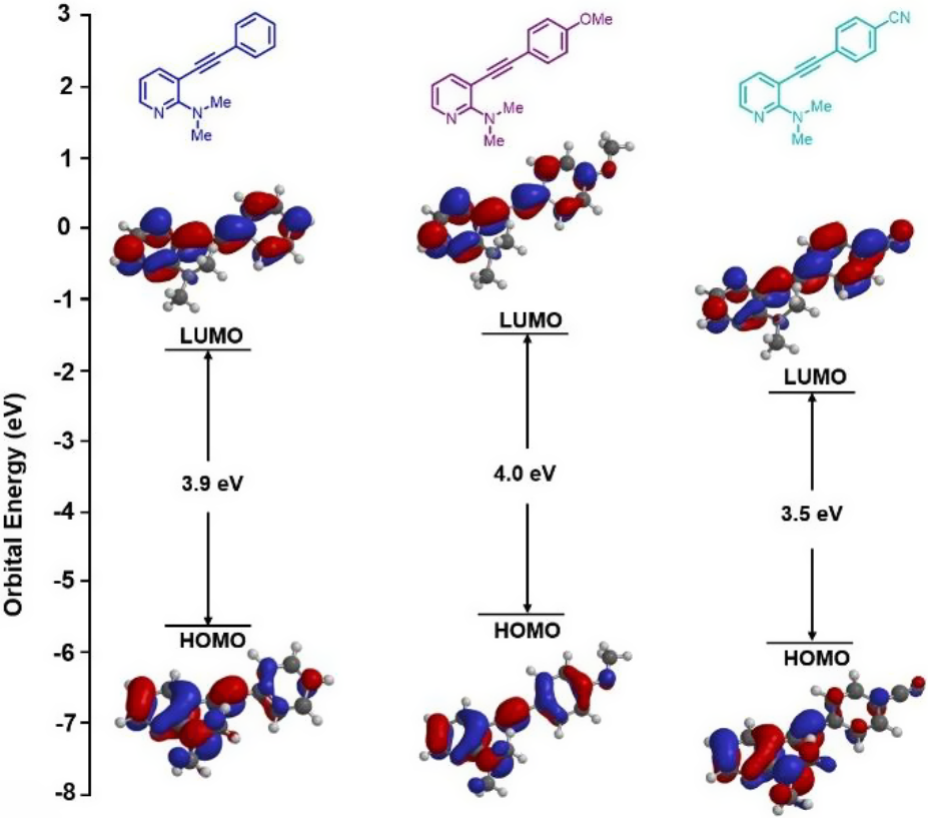
Calculated frontier orbitals and their HOMO and LUMO energies involved in electronic transition using B3LYP/6–311+G** for NNDAP and NNDAP-OMe and NNDAP-CN.

### Spectral Measurements

Figure 3 shows the normalized absorption and emission spectra for NNDAP, NNDAP-OMe, and NNDAP-CN in 10 different solvents. The peak wavelengths of the lowest-energy absorption and emission bands, absorption coefficients, Stokes shifts, and quantum yields are summarized in Table 1.

**Figure 3:**
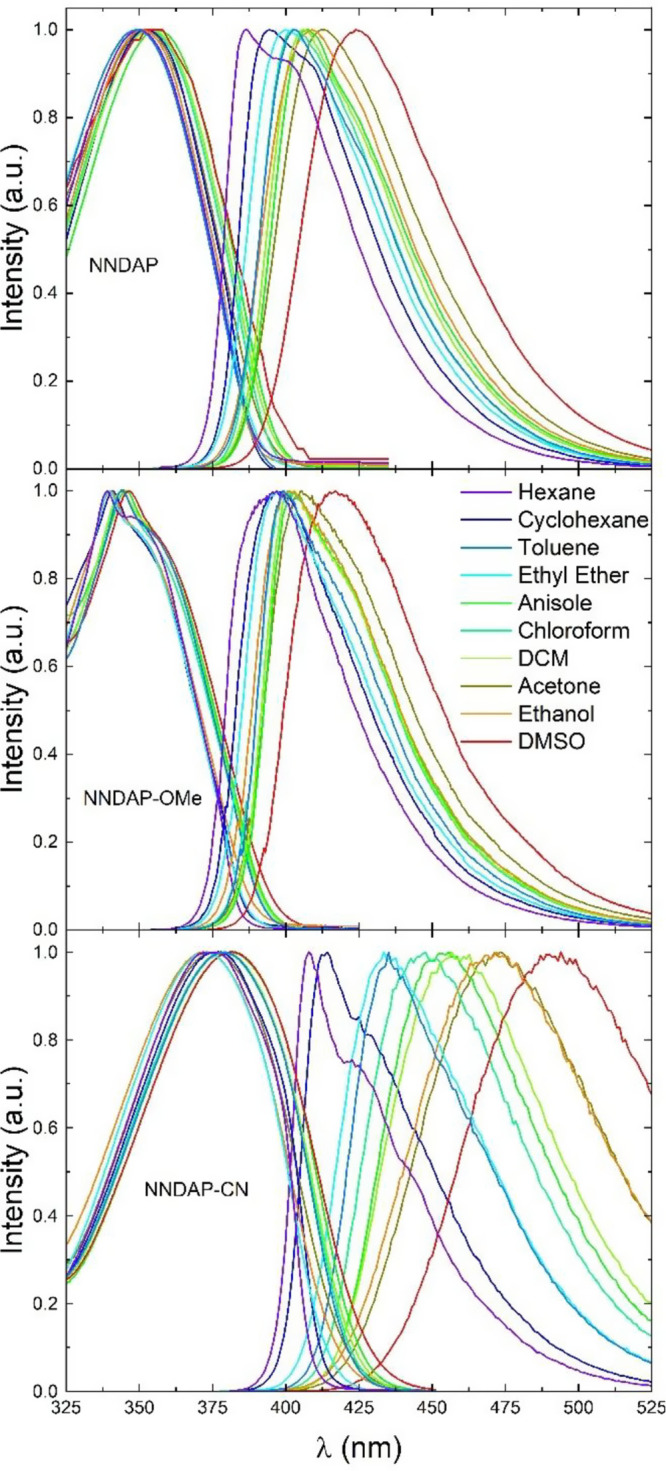
Absorption and emission spectra for NNDAP, NNDAP-OMe, and NNDAP-CN in 10 different solvents.

**Table 1:** Spectroscopic Data of NNDAP Derivatives in Different Solvents

solvent	λ_*a*_[nm] (ε_*ex*_[M^–1^ cm^–1^ ])	${\mathrm{\nu ̅}}_{a}$ [cm^–1^]	λ_*f*_[nm] (ϕ_*F*_)	${\mathrm{\nu ̅}}_{f}$ [cm^–1^]	${\mathrm{\nu ̅}}_{Stokes}$ [cm^–1^]	${\mathrm{\nu ̅}}_{a}+{\mathrm{\nu ̅}}_{f}$ [cm^–1^]
**NNDAP**						
hexane	351.0(10,692)	28,490	386.0(0.3930)	25,907	2583	54,397
cyclohexane	352.5(11,263)	28,369	394.3(0.4208)	25,361	3007	53,730
toluene	355.0(10,208)	28,169	402.9(0.6821)	24,819	3350	52,988
ethyl ether	349.0(12,162)	28,653	399.8(0.6368)	25,010	3643	53,663
anisole	356.0(11,523)	2,8090	407.5(0.9027)	24,540	33,550	52,630
chloroform	353.0(13,910)	28,329	402.9(0.6162)	24,820	3509	53,149
DCM	354.0(11,730)	28,249	406.0(0.7657)	24,631	3618	52,879
acetone	352.0(11,384)	28,409	412.0(0.2144)	24,272	4167	52,652
ethanol	349.5(9,792)	28,612	408.9(0.5613)	24,456	4156	53,068
DMSO	355.5(10,052)	28,129	423.9(0.8425)	23,590	4539	51,720
**NNDAP-OMe**						
hexane	339.00(13,997)	29,499	397.00(0.4032)	25,189	4309.6	54,687
cyclohexane	341.00(20,381)	29,326	397.00(0.6885)	25,189	4136.6	54,514
toluene	344.25(15,761)	29,049	399.00(0.5903)	25,063	3986.0	54,111
ethyl ether	339.50(15,865)	29,455	397.00(0.5041)	25,189	4266.2	54,644
anisole	345.50(18,235)	28,944	403.00(0.7886)	24,814	4129.7	53,757
chloroform	344.00(14,931)	29,070	400.50(0.2435)	24,969	4101.0	54,039
DCM	343.50(16,055)	29,112	401.50(0.6155)	24,906	4205.5	54,019
acetone	344.25(18,858)	29,049	405.25(0.6466)	24,676	4372.5	53,725
ethanol	340.00(12,803)	29,412	401.00(0.5152)	24,938	4474.1	54,349
DMSO	346.50(14,083)	28,860	417.00(0.7146)	23,981	4879.2	52,841
**NNDAP-CN**						
hexane	375.8 (15,225)	26,610	407.84 (0.5740)	24,519	2090.5	51,129
cyclohexane	377.2 (14,204)	26,511	414.15 (0.7511)	24,146	2365.3	50,657
toluene	379.0 (13,218)	26,385	435.07 (0.7125)	22,985	3400.4	49,370
ethyl ether	372.6 (14,343)	26,838	433.53 (0.7071)	23,066	3772	49,905
anisole	380.8 (12,837)	26,261	455.75 (0.7631)	21,942	4318.7	48,202
chloroform	378.8 (13,858)	26,399	447.72 (0.7110)	22,335	4063.8	48,735
DCM	378.8 (13,997)	26,399	456.60 (0.7445)	21,898	4501	48,297
acetone	375.2 (13,945)	26,653	473.18 (0.6037)	21,134	5518.8	47,786
ethanol	372.0 (13,789)	26,882	471.96 (0.5512)	21,188	5693.5	48,070
DMSO	381.0 (14,135)	26,247	493.93 (0.6242)	20,246	6000.9	46,493

### Determination of the Ground- and Excited-State Electric Dipole Moments

Solvent dielectric constants and refractive indices were used to calculate the corresponding polarity parameters, and linear regression analysis was performed to obtain model slopes. The Reichardt method was also applied using empirical solvent polarity parameters derived from betaine dye measurements. Relevant equations and solvent parameters are provided in the main text and Supporting Information. The B–K method relates the solvatochromic changes using two linear equations

$${\overset{-}{\nu }}_{\mathrm{stokes}}=m_{f}f(\epsilon ,n)+\mathrm{cons}\tan t$$ 1

$${\overset{-}{\nu }}_{A}+{\overset{-}{\nu }}_{F}=-m_{\phi }\phi (\epsilon ,n)+\mathrm{cons}\tan t$$ 2

where ${\overset{-}{\nu }}_{A}$ and ${\overset{-}{\nu }}_{F}$ are the positions of the absorption and fluorescence maxima, ${\overset{-}{\nu }}_{\mathrm{stokes}}$ is the Stokes shift, ϕ­(ϵ, *n*) = *f*(ϵ, *n*) + 2*g*(ϵ, *n*), $g\left(\epsilon ,n\right)=\frac{3\left(n^{4}-1\right)}{2{\left(n^{2}+2\right)}^{2}}$ , and $f\left(\epsilon ,n\right)=\frac{2n^{2}+1}{n^{2}+2}\left(\frac{\epsilon -1}{\epsilon +2}-\frac{n^{2}-1}{n^{2}+2}\right)$ . The slopes of eqs 1 and 2 are related to ground state and excited state dipole moments μ_*g*_ and μ_*e*_ using

$$\mu_{g}^{\mathrm{BK}}=\frac{1}{2}\left(m_{\phi }-m_{f}\right){\left(\frac{1}{2}\frac{hca^{3}}{m_{f}}\right)}^{1/2}$$ 3

$$\mu_{e}^{\mathrm{BK}}=\frac{1}{2}\left(m_{\phi }+m_{f}\right){\left(\frac{1}{2}\frac{hca^{3}}{m_{f}}\right)}^{1/2}$$ 4

where *h* is Planck’s constant, *c* is the speed of light in vacuum, *a* is the Onsager radius.^38^

The Lippert–Mataga (L–M), and Bakhshiev equations

$${\overset{-}{\nu }}_{\mathrm{stokes}}=m_{LM}F_{LM}+\mathrm{cons}\tan t$$ 5

$${\overset{-}{\nu }}_{\mathrm{stokes}}=m_{B}F_{B}+\mathrm{cons}\tan t$$ 6

where, *m*_*LM*_ (L–M), and *m*_*B*_ (Bakhshiev) are the slopes of the linear fits with the solvent parameters *F*_*LM*_ and *F*_*B*_. The solvent parameters are calculated using

$$F_{LM}\left(\epsilon ,n\right)=\frac{\epsilon -1}{2\epsilon +1}-\frac{n^{2}-1}{2n^{2}+1}$$ 7

$$F_{B}\left(\epsilon ,n\right)=\left[\frac{\epsilon -1}{\epsilon +1}-\frac{n^{2}-1}{2n^{2}+1}\right]\frac{2n^{2}+1}{n^{2}+1}$$ 8

where ϵ is the solvent dielectric constant and *n* is the solvent index of refraction. The slopes are related to the difference between the excited and ground state dipole moments by

$$\Delta \mu^{LM}=\sqrt{\frac{1}{2}m_{LM}hca^{3}}$$, 9

$$\Delta \mu^{B}=\sqrt{\frac{1}{2}m_{B}hca^{3}}$$. 10

Reichardt used betaine dyes^35,39,40^ as probes and experimentally developed an extensive table of values of a nonspecific solvent polarity parameter *E*_*T*_^*N*^. This model correlates the values of the shift in absorption and fluorescence peaks to the solvent parameter using eqs 11 and 12.

$${\overset{-}{\nu }}_{\mathrm{stokes}}=11\text{,}307.6\left[\left(\frac{\Delta \mu^{2}}{\Delta \mu_{B}}\right){\left(\frac{a_{B}}{a_{0}}\right)}^{2}\right]E_{T}^{N}+\mathrm{cons}\tan t$$ 11

$${\overset{-}{\nu }}_{\mathrm{stokes}}=m_{R}E_{T}^{N}+\mathrm{cons}\tan t$$ 12

where Δμ_*B*_ = 9 D and *a*_*B*_ = 6.2 Å are the change in dipole moment upon excitation and the Onsager radius of the reference betaine dye, respectively.

The slope of the line fit is associated with the difference between the ground and excited state electric dipole moments by eq 13.

$$\Delta \mu^{R}=\mu_{\mathrm{ex}}-\mu_{g}=\sqrt{\frac{81m_{R}}{11\text{,}307.6{\left(\frac{6.2}{a}\right)}^{3}}}$$ 13

Scatter plots of the solvatochromic data summarized in Table 1 as a function of solvent polarity parameters for NNDAP, NNDAP-OMe, and NNDAP-CN are shown in Figure 4. Linear regression was performed for each correlation model to determine the slopes associated with the Bilot–Kawski, Lippert–Mataga, Bakhshiev, and Reichardt methods. All statistically significant fits exhibited *p*-values < 0.05 based on ANOVA analysis, except for selected parameters for NNDAP-OMe, where weaker solvent dependence was observed.

**Figure 4:**
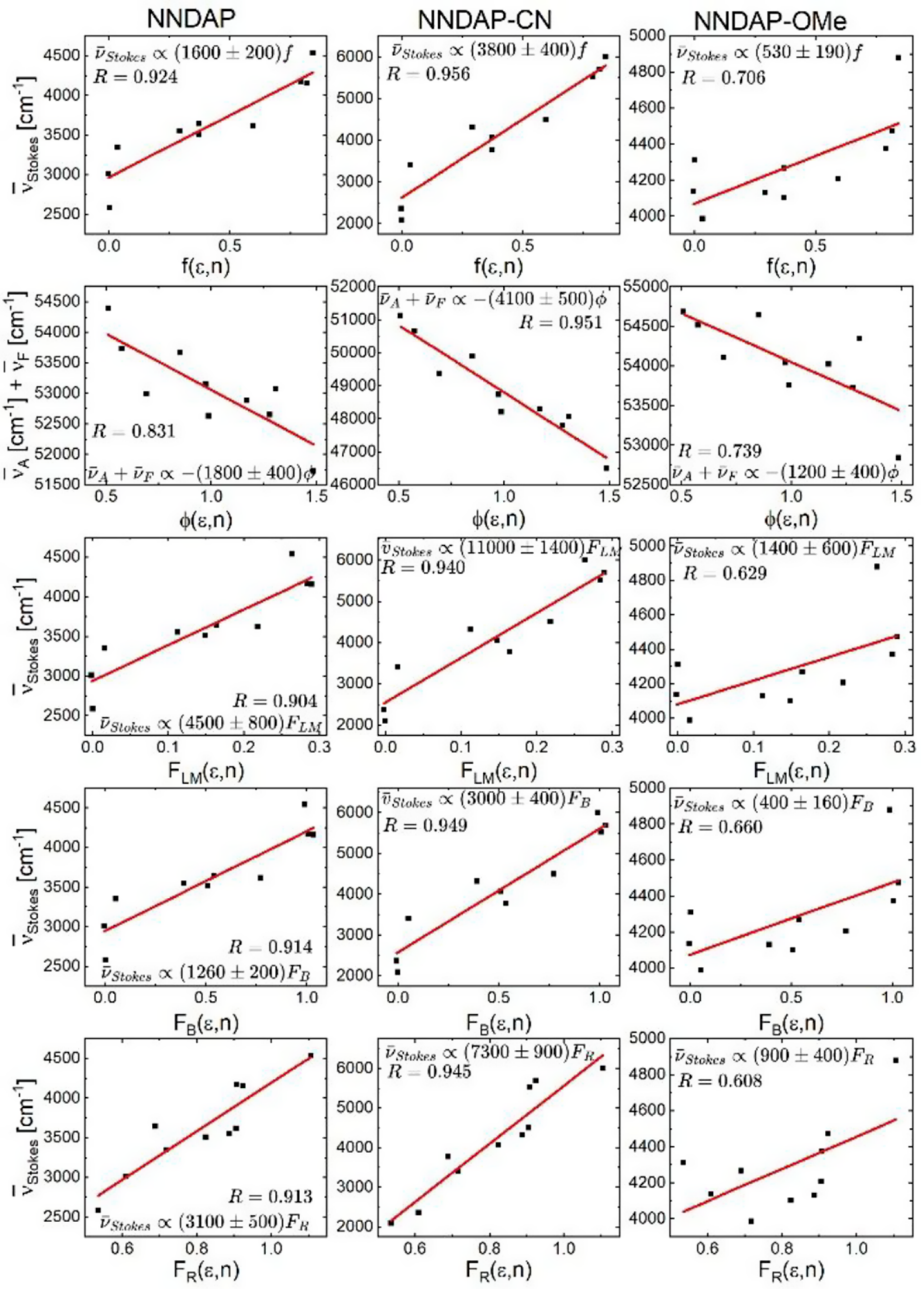
Linear plots of the Bilot–Kawski, Lippert–Mataga, Bakhshiev, and Reichardt correlation methods for NNDAP, NNDAP-OMe, and NNDAP-CN.

The slopes obtained from the statistically significant fits were used to calculate the ground-state (μ_*g*_), excited-state (μ_*e*_), and change in dipole moment (Δμ) for each fluorophore using eqs 3, 4, 9, 10, and 13. A summary of the calculated dipole moments is presented in Table 2. Consistent with previous reports, including the work of Manohara et al., the Lippert–Mataga method yields systematically larger values of Δμ compared to other models, reflecting its neglect of solute polarizability effects.^30^

**Table 2:** Ground and Singlet Excited State Dipole moments (Debye) of NNDAP, NNDAP-OMe and NNDAP-CN for Bilot–Kawski, Lippert–Mataga, Bakhshiev, Reichardt Correlation Methods for an Onsager Radius 4.29 Å

sample	μ_*g*_^*BK*^	μ_*e*_^*BK*^	Δμ^*LM*^	Δμ^*B*^	Δμ^R^
NNDAP	0.3 ± 0.5	3.8 ± 0.5	5.943 ± 0.018	3.147 ± 0.018	2.7 ± 0.2
NNDAP-OMe	1.4 ± 0.8	3.4 ± 0.8		1.77 ± 0.03	
NNDAP-CN	0.2 ± 0.4	5.7 ± 0.4	9.226 ± 0.016	4.882 ± −0.015	4.2 ± 0.3

The Bilot–Kawski analysis indicates small to negligible ground-state dipole moments for NNDAP and NNDAP-CN, while revealing substantial excited-state dipole moments for all three compounds. This behavior indicates increased polarity upon excitation, consistent with charge redistribution in the excited state. Among the three fluorophores, NNDAP-CN exhibits the largest change in dipole moment, suggesting enhanced excited-state charge separation relative to NNDAP and NNDAP-OMe. This increased polarity is expected to strengthen solute–solvent interactions and contribute to the pronounced solvatochromic response observed for NNDAP-CN. Overall, the magnitude of the excited-state dipole moment increase follows the substituent trend NNDAP-OMe < NNDAP < NNDAP-CN, consistent with increasing electron-withdrawing character and enhanced charge redistribution in the excited state.

### Multi-Parameter Linear Solvation Energy Relationship Analysis

To further elucidate the origin of solvent-dependent spectral shifts, the absorption and emission maxima were analyzed using the Kamlet–Taft^36^ and Catalán^37^ multiparameter linear solvation energy relationship (LSER) models using OriginPro. Regression analyses were performed using literature solvent parameters (provided in the Supporting Information), and statistical significance was evaluated by analysis of variance (ANOVA) with a threshold of *p* < 0.05. The significance of the model as a whole is presented using the *F*–test­(*Pr* > *F*). If the model is significant (<0.05), then the *t*–test (*Pr* < |*t*|) identifies the individual variables of significance (<0.05). A summary of these results in presented in Table 3.

**Table 3:** Multi-parameter Linear Regression Analysis of NNDAP, NNDAP-OMe, and NNDAP– CN

	NNDAP		NNDAP-OMe		NNDAP-CN	
	abs(*P* > |t|)	emis (*P* > |t|)	abs(*P* > |t|)	emis (*P* > |t|)	abs(*P* > |t|)	emis (*P* > |t|)
Kamlet–Taft						
Pr > F	0.030	<0.001	0.007	0.022	0.057	<0.001
multiple- R	0.867	0.970	0.861	0.934	0.831	0.983
*C*_α_	303 (0.128)	–2.56 (0.993)	253 (0.157)	–462 (0.176)	315 (0.200)	–611 (0.207)
*C*_β_	289 (0.171)	–868 (0.022)	166 (0.366)	–629 (0.102)	383 (0.158)	–1576 (0.015)
*C*_π*_	–473 (0.015)	–1228 (0.001)	–614 (0.002)	–594 (0.053)	–504 (0.031)	–2866 (<0.001)
Catalán						
Pr > F	0.007	0.002	0.006	0.068	0.003	<0.001
multiple- R	0.956	0.973	0.959	0.883	0.968	0.994
*C*_*SP*_	–2073 (0.003)	–2528 (0.041)	–1848 (0.007)	–1969 (0.140)	–2301 (0.001)	–3238 (0.020)
*C*_*SdP*_	29.3 (0.818)	–877 (0.032)	–276 (0.098)	–369 (0.352)	–16.3 (0.894)	–2775 (<0.001)
*C*_*SA*_	371 (0.196)	–0.734 (0.999)	479 (0.147)	–634 (0.430)	341 (0.215)	–863 (0.231)
*C*_*SB*_	–9.79 (0.956)	–1069 (0.051)	–15.5 (0.938)	–723 (0.212)	118 (0.505)	–1206 (0.039)

The Kamlet–Taft model describes solvatochromic behavior in terms of nonspecific dipolarity/polarizability (π*) and specific hydrogen-bond donor (α) and acceptor (β) interactions^36^

$$\overset{-}{\nu }=C_{\pi *}\pi^{*}+C_{\alpha }\alpha +C_{\beta }\beta +C_{0}$$ 14

For the absorption spectra of NNDAP and NNDAP-OMe, the dominant statistically significant contribution arises from π*, indicating that ground-state electronic transitions are governed primarily by nonspecific dipolar stabilization. In contrast, the emission spectra of NNDAP and NNDAP-CN show significant dependence on both π* and β, demonstrating that excited-state solute–solvent interactions involve not only bulk dipolarity but also solvent basicity. The comparatively weaker solvatochromic response of NNDAP-OMe is reflected in reduced statistical significance of its β contribution in emission.

Because Kamlet–Taft coefficients are not normalized and therefore limit direct comparison of interaction magnitudes, the Catalán model was applied to independently resolve solvent polarizability (SP) and dipolarity (SdP), along with solvent acidity (SA) and basicity (SB) contributions.

$$\overset{-}{\nu }=C_{SP}SP+C_{SdP}SdP+C_{SA}SA+C_{SB}SB+C_{0}$$ 15

The Catalán analysis reveals that incorporation of the electron-donating methoxy substituent decreases the susceptibility of the fluoropherto induced dipole interactions, whereas introduction of the electron-withdrawing nitrile group substantially enhances solvent–solute dipolar interactions. In the emission data, increased sensitivity to SdP confirms greater excited-state dipole moment relative to the ground state, consistent with the solvatochromic and dipole moment analyses.

For NNDAP-CN, a statistically significant SB term is observed in the excited state, with a similar trend approaching significance for NNDAP. This behavior indicates that solvent basicity contributes to stabilization of the excited state, whereas the ground-state absorption transition is primarily influenced by nonspecific dipolar effects. The increased sensitivity to both solvent dipolarity and basicity following excitation is consistent with enhanced charge redistribution in the excited state. Such behavior is characteristic of fluorophores exhibiting intramolecular charge-transfer character, where increased excited-state polarity promotes stronger interactions with polar and basic solvents. Collectively, the LSER analyses indicate that solvent effects in this fluorophore series are dominated by dipolar stabilization, with additional contributions from specific solute–solvent interactions in the excited state.

Overall, the solvatochromic dipole moment analysis, LSER regression models, and density functional theory calculations collectively support a consistent mechanistic interpretation of the excited-state behavior in the NNDAP fluorophore series. Bilot–Kawski and Bakhshiev analyses indicate a significant increase in dipole moment upon excitation, suggesting enhanced excited-state polarity. Consistent with this observation, the Catalán LSER analysis shows increased sensitivity of the emission spectra to solvent dipolarity and basicity parameters, indicating stronger solvent stabilization of the excited state. Density functional theory calculations further support this interpretation, revealing systematic stabilization of the LUMO with increasing electron-withdrawing character of the substituent, particularly for NNDAP-CN. Together, these results indicate that excitation promotes charge redistribution across the π-system, producing an excited state with greater polarity and stronger interactions with polar solvents.

### Fluorescence visualization in HeLa cells

The favorable photophysical characteristics of the NNDAP fluorophore series prompted evaluation of their behavior in live-cell fluorescence imaging. All three compounds exhibit blue emission, with excitation and emission maxima centered near 357 and 447 nm, respectively, (Figure 5). Fluorescence was localized predominantly within the cytoplasm, with enhanced intensity in the perinuclear region and minimal nuclear staining. Differences in emission intensity were observed across the series, with NNDAP displaying the weakest signal and NNDAP-CN exhibiting the strongest fluorescence and most defined localization. These trends are consistent with the greater excited-state polarity and enhanced solvatochromic sensitivity observed for NNDAP-CN. Although no specific organelle targeting is implied, the observed cellular uptake and fluorescence stability indicate that these fluorophores may be suitable as environment-responsive imaging probes.

**Figure 5:**
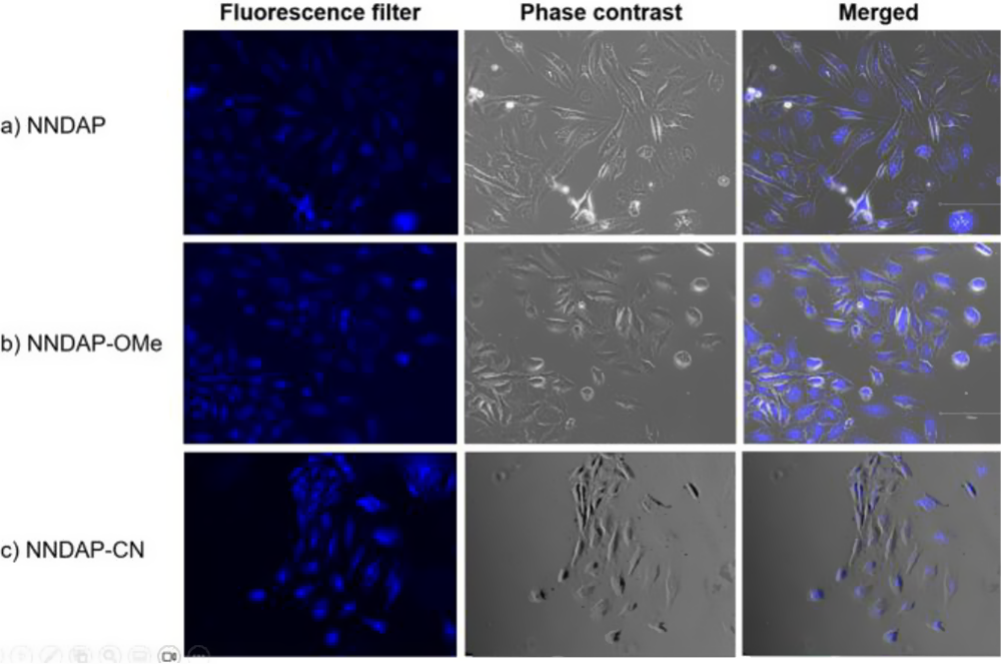
Fluorescence visualization of HeLa cells after 15 min incubation with 100 μg/mL (in PBS) of (a) NNDAP (b) NNDAP-OMe and (c) NNDAP-CN compounds using fluorescence filter with excitation: 357 nm and emission: 447 nm, phase contrast and overlay/merged image at 10× magnification.

## Conclusions

The solvent-dependent absorption and fluorescence behavior of three pyridine-based fluorophores—NNDAP, NNDAP-OMe, and NNDAP-CN—was systematically investigated using classical solvatochromic correlation methods together with multiparameter linear solvation energy relationship (LSER) models. Analysis using the Bilot–Kawski, Lippert–Mataga, Bakhshiev, and Reichardt formalisms indicates that all three compounds undergo an increase in dipole moment upon excitation, with NNDAP-CN exhibiting the largest change in electric dipole moment. This trend correlates with the electron-withdrawing strength of the substituent and the pronounced solvatochromic response observed for the nitrile-substituted derivative.

Multiparameter regression using the Kamlet–Taft Solvatochromic Parameters and Catalán Solvent Parameter Model shows that absorption spectral shifts are governed primarily by nonspecific solvent dipolarity/polarizability effects, whereas emission shifts display additional sensitivity to solvent dipolarity and basicity. These results indicate that the excited states of the fluorophores experience enhanced stabilization in polar environments, consistent with increased charge redistribution following excitation. The Catalán analysis further demonstrates that substituent electronic effects modulate the strength of these solvent interactions, with the electron-withdrawing nitrile group promoting stronger excited-state polarity and solvent stabilization.

All three fluorophores readily permeate HeLa cells and produce stable blue fluorescence localized primarily within the perinuclear cytoplasmic region. Collectively, these findings demonstrate that substituent-controlled modulation of frontier orbital energies translates directly into tunable excited-state dipole moments and solvent-dependent fluorescence behavior, establishing a rational framework for the design of environment-sensitive fluorophores.

## Supplementary Material



## Notes

Additional resources including the synthesis of NNDAP molecules, corresponding ^1^H and ^13^C NMR spectra, and solvent parameters used in the manuscript (PDF)
