# Supplementary material for: Solvatochromic Study of 2‑(N,N‑dimethyl)-3-alkynyl-pyridine Fluorophores
Source: ACS Omega. 2026 Jun 22;11(25):38106–13. doi: 10.1021/acsomega.6c03632 (PMC13325342; doi:10.1021/acsomega.6c03632)
Supplement: Supplementary file 1 [file ao6c03632_si_001.pdf]

Solvatochromic study of 2-(N,N-dimethyl)-3-alkynyl-pyridine fluorophores

Leah Gross[1], Aimee Phillips[1], Lavender Allen[1], Zane Burnett[1], Korinna Welch[1], Prerna Masih[2], Chandra Prayaga[1], Tanay Kesharwani[3*] and Aaron Wade[1]*

[1]*Department of Physics, University of West Florida, Pensacola, FL, USA*
2Department of Biology, Bard College, Annandale-on-Hudson, NY, USA
3Department of Chemistry, Bard College, Annandale-on-Hudson, NY, USA

Table of Contents

	**Pages**
General.	S2-S2
Reagents.	S2-S2
Procedure for the synthesis of 3-iodo-*N,N*-dimethyl-pyridin-2-amine	S2-S2
General procedure for the synthesis of compounds NNDAP, NNDAP-CN and NNDAP-OMe	S2-S4
^{1}H and ^{13}C NMR spectra.	S4-S7
Solvent Parameters	S8

General. The ^{1}H and ^{13}C NMR spectra were recorded on 400 and 100 MHz Bruker NMR. High-resolution mass spectra (HRMS) were verified on a VG-70S magnetic sector mass spectrometer using a direct probe sample introduction and electron ionization (EI). Thin layer chromatography was performed using commercially prepared 60-mesh silica gel plates, and visualization was affected with short and long wavelength UV light. All melting points are uncorrected.

Reagents. All reagents were obtained commercially unless otherwise noted.

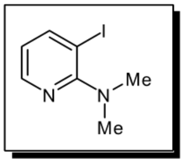

Procedure for the synthesis of 3-iodo-*N,N*-dimethyl-pyridin-2-amine. To a three-neck round bottom flask, 2-fluoro-3-iodopyridine (22.0 mmol) was dissolved in 44 mL DMF. The reaction refluxed at 95 °C with strong stirring. An aqueous KOH solution (5.0 equiv, 10 M) was added in 30-minute intervals until reaction completion. The reaction was supervised via TLC and gas chromatography. After usual workup procedure the reaction mixture was purified via column chromatography using varying concentrations of hexanes and ethyl acetate as an eluent. Product was isolated as a yellow oil; ^{1}H NMR (400 MHz, $CDCl_3$) δ 2.89 (s, 6H), 6.51 (dd, *J* = 7.6, 4.8 Hz, 1H), 7.96 (dd, *J* = 7.6, 1.6 Hz, 1H), 8.17 (dd, *J* = 4.8, 1.6 Hz, 1H); ^{13}C NMR (100 MHz, $CDCl_3$) δ 42.96, 86.06, 118.45, 146.92, 149.27, 163.28; IR (neat, cm^{-1}) 3041, 2995, 2943, 2841, 2791, 1895, 1728, 1574, 1481, 1401, 1333, 1244, 1109, 999, 955, 782, 641; HRMS (EI^+, m/z) calcd for $(C_7H_9N_2I)^+$ 247.9810, found 247.9814.

General procedure for the synthesis of starting compounds (NNDAP and NNDAP-CN). To a reaction flask, 3-iodo-*N,N*-dimethylpyridin-2-amine (1.47 mmol) was added with the corresponding terminal alkyne (1.20 equiv.) was added. Triethylamine (5 mL) was then added followed by Copper(I) iodide (0.15 mmol) and $PdCl_2(PPh_3)_2$ (0.07 mmol). The mixture stirred at ambient conditions for 24 hours. The reaction mixture was purified via column chromatography using varying concentrations of hexanes and ethyl acetate as an eluent.

***N,N*-dimethyl-3-(2-phenylethynyl)pyridin-2-amine (NNDAP).** Product was isolated as a yellow oil; ^{1}H NMR (400 MHz, $CDCl_3$) δ 3.24 (s, 6H), 6.66 (dd, *J* = 7.6, 4.8 Hz, 1H), 7.31-7.39 (m, 3H), 7.47-7.54 (m, 2H), 7.66 (dd, *J* = 7.6, 2.0 Hz, 1H), 8.15 (dd, *J* = 5.2, 2.0 Hz, 1H); ^{13}C NMR (100 MHz, $CDCl_3$) δ 41.35, 88.21, 95.63, 106.20, 113.83, 123.83, 128.58, 128.75, 131.38, 143.12, 147.38, 161.59; IR (neat, cm^{-1})

3058, 2942, 2791, 2208, 1579, 1551, 1491, 1401, 1239, 960, 756, 690; HRMS (EI$^+$, m/z) calcd for $(C_{15}H_{14}N_2)^+$ 222.1157, found 222.1153.

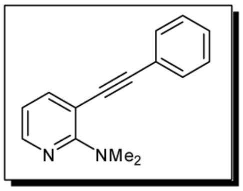

4-[2-[2-(dimethylamino)-3-pyridyl]ethynyl]benzonitrile (NNDAP-CN). Product was isolated as a green/yellow solid; mp = 67-69; ^{1}H NMR (400 MHz, $CDCl_3$) δ 3.23 (s, 6H), 6.65 (dd, J = 7.6, 4.8 Hz, 1H), 7.54 (d, J = 8.4, 2H), 7.61 (d, J = 8.8, 2H), 7.65 (dd, J = 7.6, 2.0 Hz, 1H), 8.17 (dd, J = 4.8, 2.0 Hz, 1H); ^{13}C NMR (100 MHz, $CDCl_3$) δ 41.24, 93.02, 93.76, 104.45, 111.56, 113.67, 118.81, 128.71, 131.65, 132.39, 143.45, 148.36, 161.44; IR (neat, cm^{-1}) 2923, 2351, 2347, 1578, 1401, 841, 787, 721; HRMS (EI$^+$, m/z) calcd for $(C_{16}H_{13}N_3)^+$ 222.1157, found 222.1153.

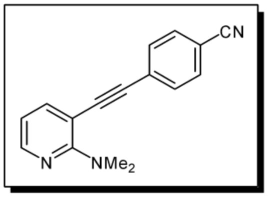

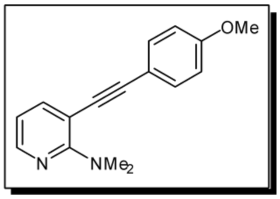

3-[2-(4-Methoxyphenyl)ethynyl]-*N,N*-dimethyl-pyridin-2-amine (NNDAP-OMe). The product was isolated as a yellow solid; mp = 72-75; ^{1}H NMR (400 MHz, $CDCl_3$) δ 3.22 (s, 6H), 3.82 (s, 3H).6.65 (dd, J= 7.6, 4.8, 1H)6.87 (d, J= 9.2, 2H), 7.43 (d, J= 8.8, 2H), 7.64 (dd, J= 7.2, 2.0, 1H), 8.13 (dd, J= 4.8, 1.6, 1H); ^{13}C NMR (100 MHz, $CDCl_3$) δ 41.18, 55.49, 86.65, 95.53, 113.79, 114.24, 115.81, 132.70, 142.60, 146.94, 159.81, 161.52; IR (neat, cm^{-1}) 3429, 1650, 1508, 1400, 1244, 1030, 834, 764; HRMS (EI$^+$, m/z) calcd for $(C_{16}H_{16}N_2O)^+$ 252.1262, found 252.1267.

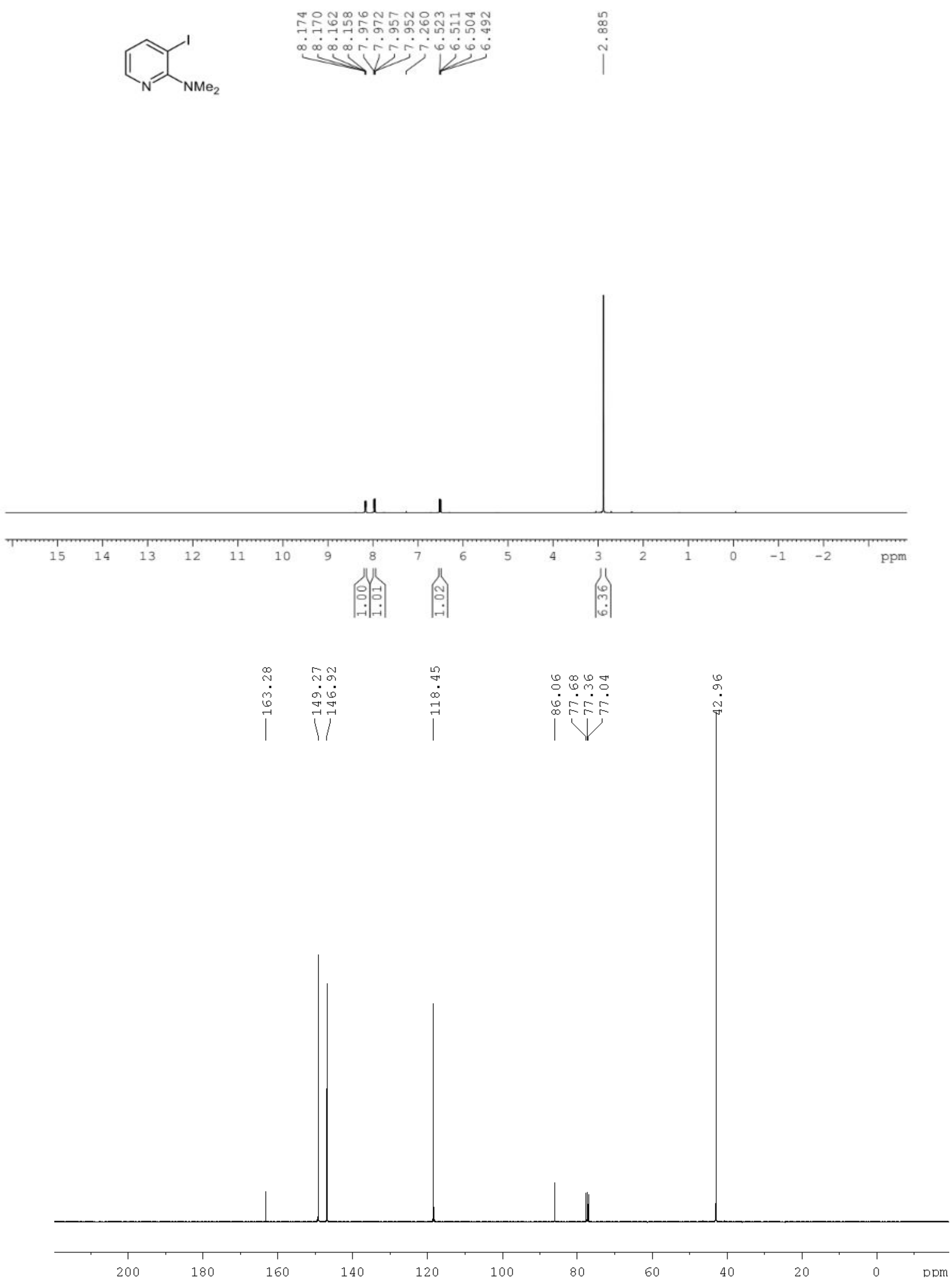

Figure S1. ^{1}H and ^{13}C NMR of 2-Iodo-*N,N*-dimethylpyridin-2-amine

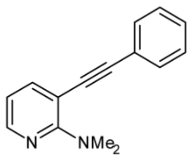

NNDAP

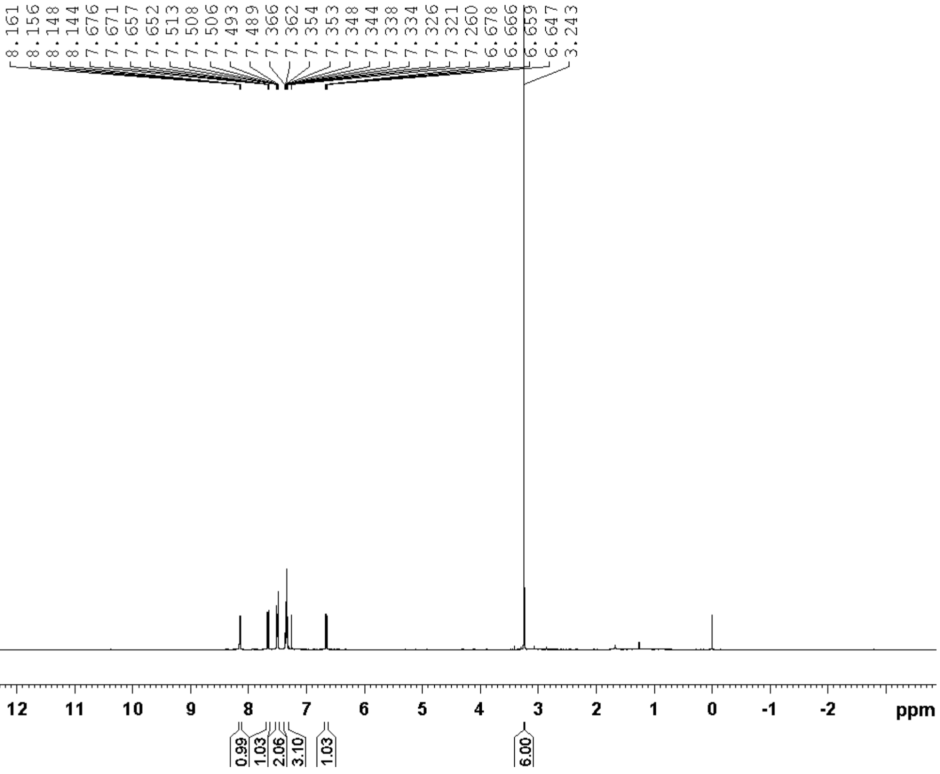

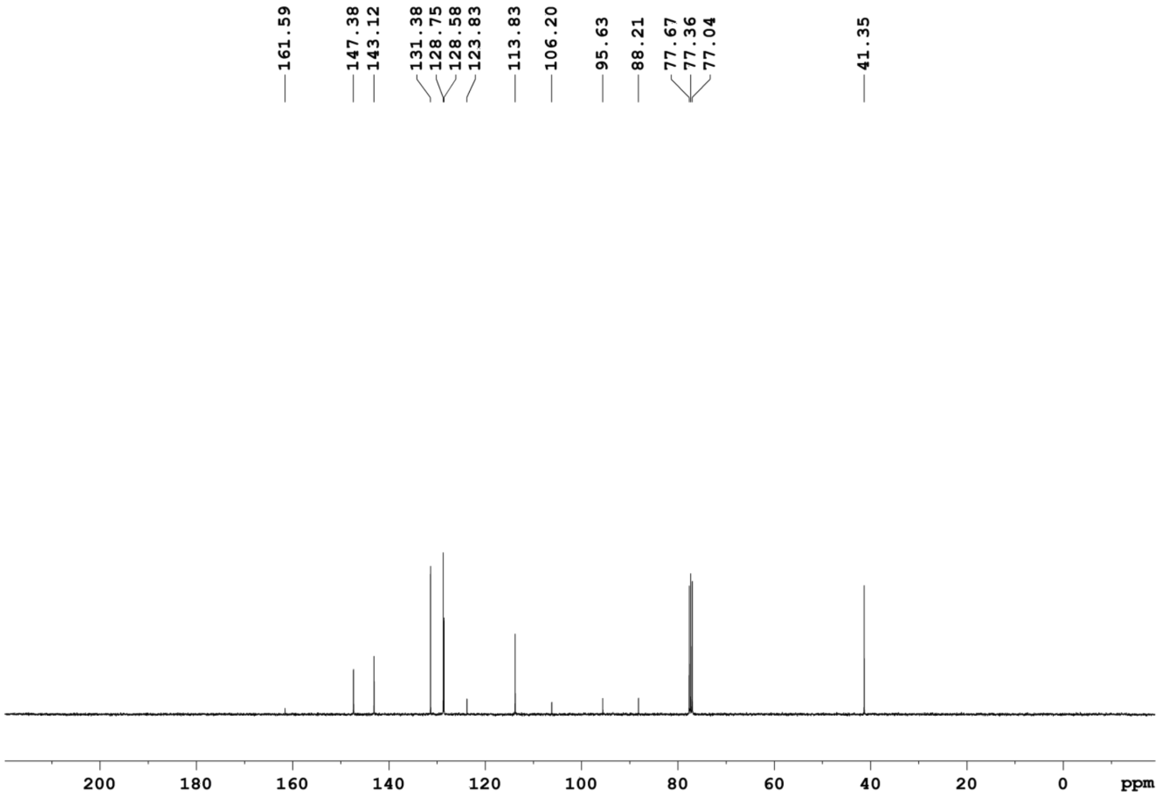

Figure S2. ^{1}H and ^{13}C NMR of NNDAP

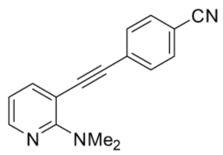

NNDAP-CN

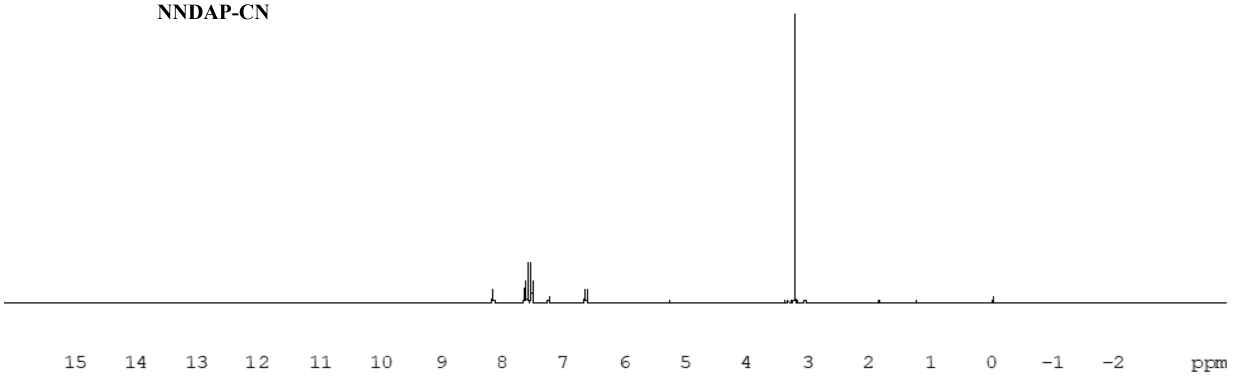

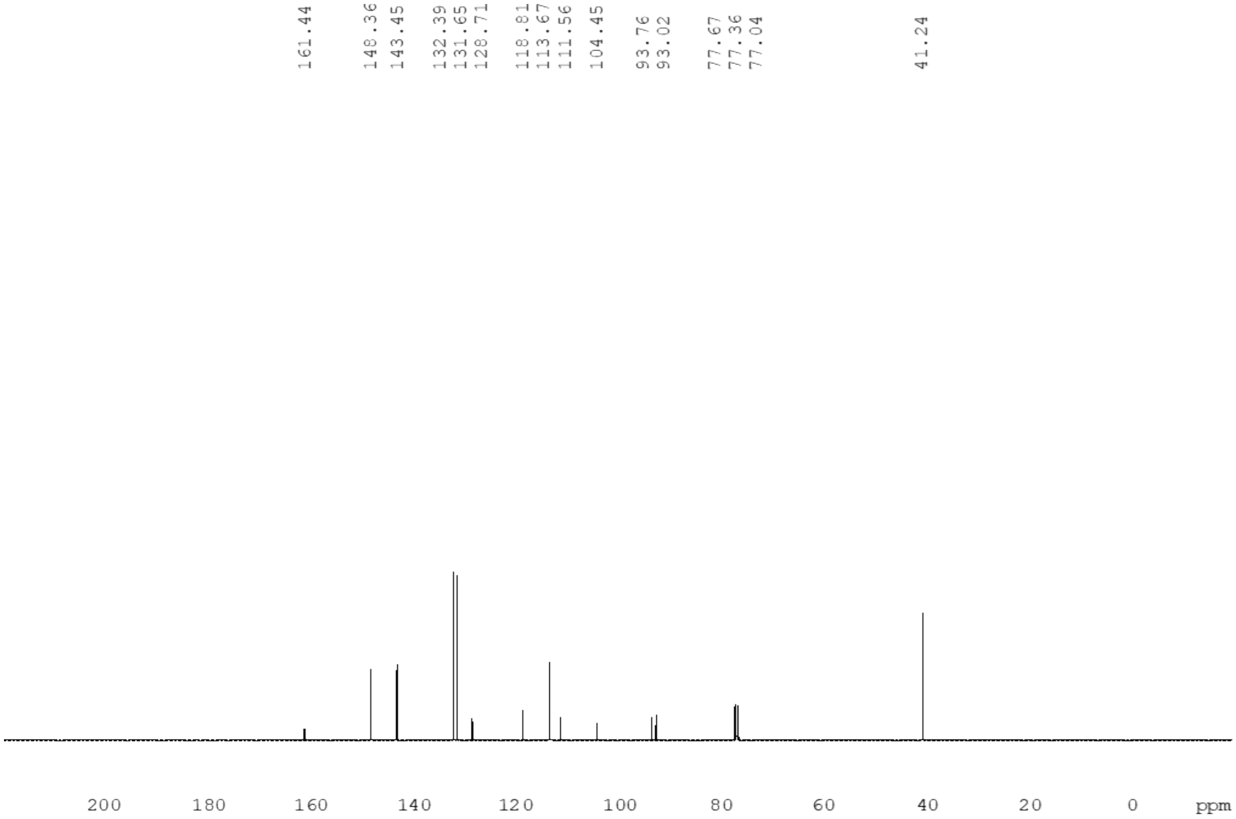

Figure S3. ^{1}H and ^{13}C NMR of NNDAP-CN

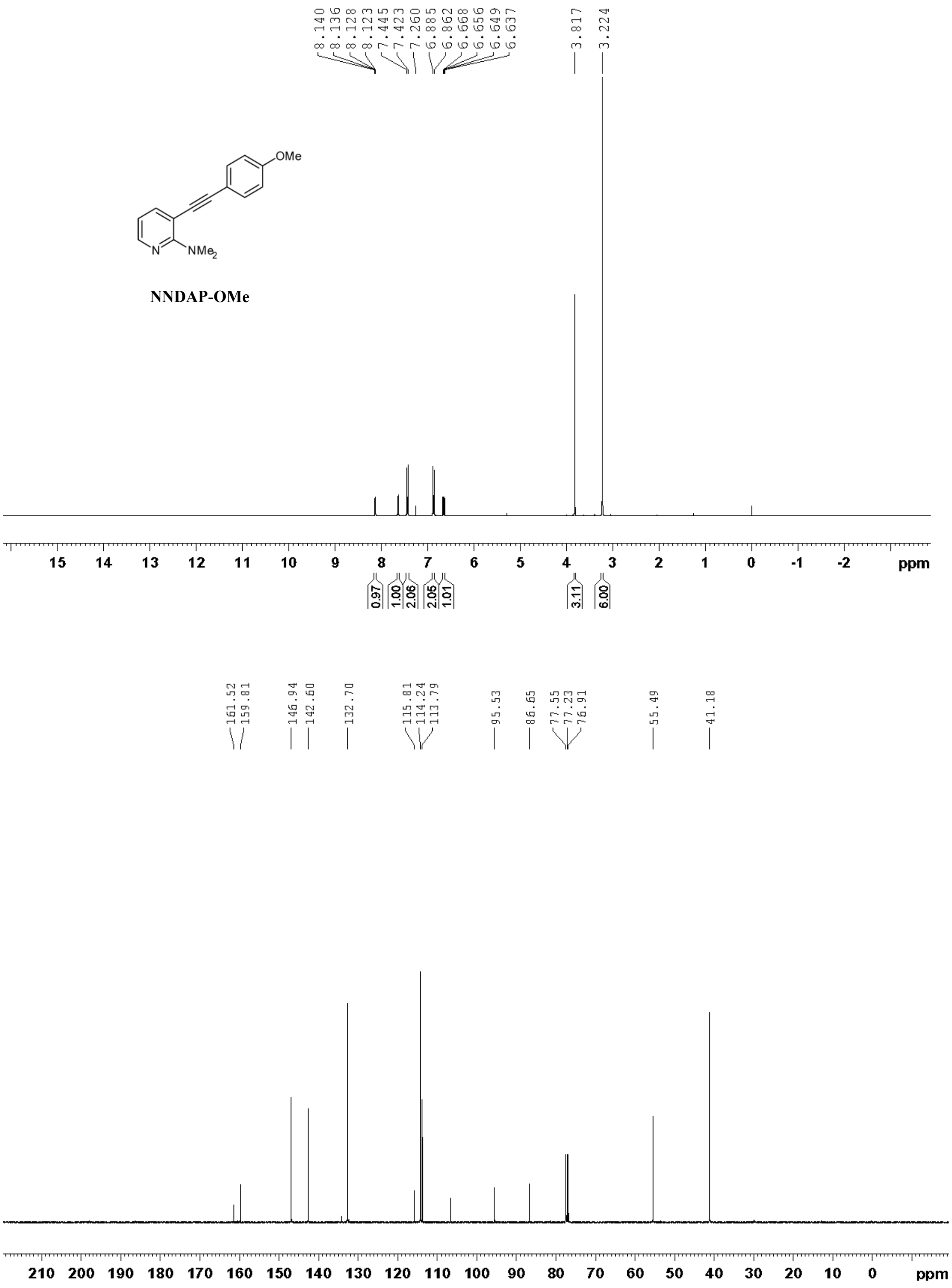

Figure S4. ^{1}H and ^{13}C NMR of NNDAP-OMe

Solvent Parameters

Table S1 Calculated values for solvent polarity functions using Lippert– Mataga $F_1(\varepsilon,n)$, Bakhshiev $F_2(\varepsilon,n)$, dielectric constant ε, refractive index n, Reichardt, and Bilot–Kawski methods

**Solvent**	$F_{LM}(\varepsilon,n)$	$F_B(\varepsilon,n)$	ε	n	E_T^N	$f(\varepsilon,n)$	$\phi(\varepsilon,n)$
Acetone	0.284	1.007	4.33	1.517	0.198	0.791	1.279
Anisole	0.112	0.391	4.81	1.445	0.250	0.291	0.988
Chloroform	0.149	0.509	2.02	1.4262	0.006	0.371	0.975
Cyclohexane	-0.00165	-0.00512	9.08	1.424	0.310	-0.00326	0.575
DCM	0.218	0.772	47.24	1.477	0.440	0.595	1.171
DMSO	0.264	0.990	25.33	1.361	0.650	0.842	1.487
Ethanol	0.290	1.031	4.27	1.353	0.110	0.817	1.308
Ethyl Ether	0.165	0.539	1.89	1.374	0.000	0.371	0.851
Hexane	2.79E-4	8.417E-4	2.38	1.487	0.090	5.175E-4	0.509
Toluene	0.0162	0.0523	4.33	1.517	0.198	0.0354	0.693

Table S2 Kamlet-Taft and Catalan solvent parameters

	**Kamlet-Taft**			**Catalan**			
**solvent**	α	β	π^*	**SP**	**SdP**	**SA**	**SB**
C_3H_6O, Acetone	0.08	0.43	0.71	0.651	0.907	0	0.475
C_7H_8O, Anisole	0	0.32	0.73	0.82	0.543	0.084	0.299
$CHCl_3$, Chloroform	0.2	0.1	0.53	0.783	0.614	0.047	0.071
C_6H_{12}, Cyclohexane	0	0	0	0.683	0	0	0.073
CH_2Cl_2, Dichloromethane	0.13	0.1	0.82	0.761	0.769	0.04	0.178
C_2H_6OS, DMSO	0	0.76	1	0.83	1	0.072	0.647
C_2H_6O, Ethanol	0.86	0.75	0.54	0.633	0.783	0.4	0.658
$C_4H_{10}O$, Ethyl Ether	0	0.47	0.27	0.617	0.385	0	0.562
C_6H_{14}, Hexane	0	0	-0.04	0.616	0	0	0.056
C_7H_8, Toluene	0	0.11	0.54	0.782	0.284	0	0.128
